# Supplementary material for: Combination of ethyl acetate fraction from Calotropis gigantea stem bark and sorafenib induces apoptosis in HepG2 cells
Source: PLoS One. 2024 Mar 25;19(3):e0300051. doi: 10.1371/journal.pone.0300051 (PMC10962855; doi:10.1371/journal.pone.0300051)
Supplement: S1 Fig — (PDF) [file pone.0300051.s001.pdf]

Supporting information

S1 Fig. The high-pressure liquid chromatography-electrospray ionisation-mass spectroscopic (HPLC-ESI-MS) chromatogram of *C. gigantea* stem bark extracts (CGEtOAc) in negative mode.

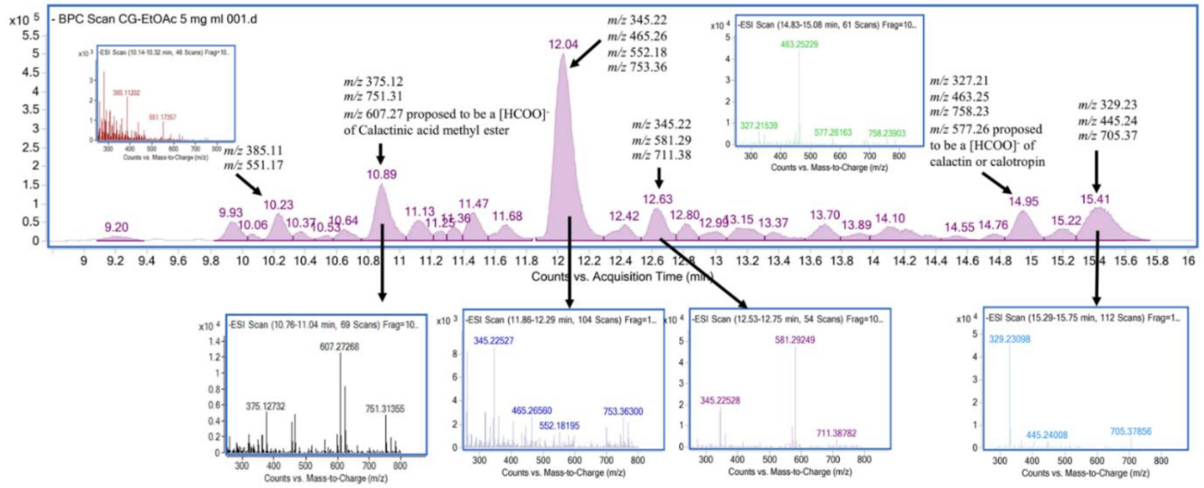

The HPLC-ESI-MS chromatogram of CGEtOAc was obtained from an Agilent 6540 UHD LCMS instrument. CGEtOAc was dissolved in methanol (MS grade, 5 mg/mL, 10 μL) then injected into a stationary phase using a Phenomenex Luna® 3 μm C18 column (150 mm × 4.6 mm i). The temperatures of autosampler and column were 4 and 25 °C, respectively. A mobile phase was a gradient solution of 0.1% formic acid-water solution and 0.1% formic acid-acetonitrile solution (5–95% acetonitrile, 20 min, 0.8 mL/min flow rate). The electrospray ionization mass spectrometry in negative mode (m/z range 200–800) setup with a 30 psi nebulizer pressure (N2), a 10 L/min drying gas flow rate, and a 350 °C temperature was performed. The retention time in the base peak chromatogram (BPC) and mass spectra of possible m/z of CGEtOAc were illustrated.
