# Supplementary material for: Combination of ethyl acetate fraction from Calotropis gigantea stem bark and sorafenib induces apoptosis in HepG2 cells
Source: PLoS One. 2024 Mar 25;19(3):e0300051. doi: 10.1371/journal.pone.0300051 (PMC10962855; doi:10.1371/journal.pone.0300051)

Supporting information

S2 Fig. The IC_{50} curves for (A) CGEtOAc and (B) sorafenib in HepG2 cells, and (C) CGEtOAc in IMR-90 cells for 24 h of incubation.

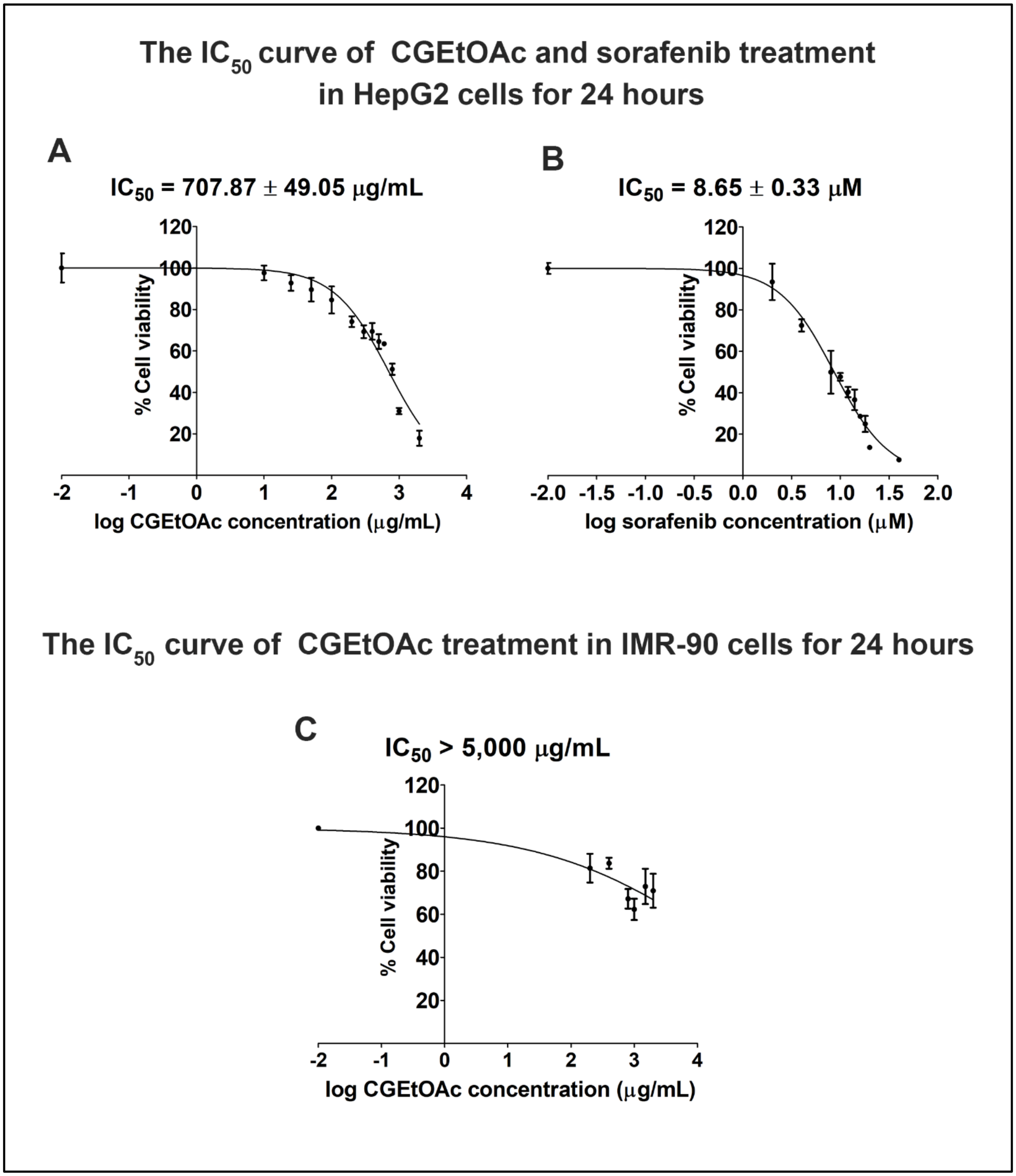

Supplement: S2 Fig — (PDF) [file pone.0300051.s002.pdf]
