# Supplementary material for: Combination of ethyl acetate fraction from Calotropis gigantea stem bark and sorafenib induces apoptosis in HepG2 cells
Source: PLoS One. 2024 Mar 25;19(3):e0300051. doi: 10.1371/journal.pone.0300051 (PMC10962855; doi:10.1371/journal.pone.0300051)
Supplement: S3 Fig — (PDF) [file pone.0300051.s003.pdf]

Supporting information

S3 Fig. The combination index (CI) vs. fraction affected (Fa) graph for CGEtOAc in combination with sorafenib after 24 h of incubation.

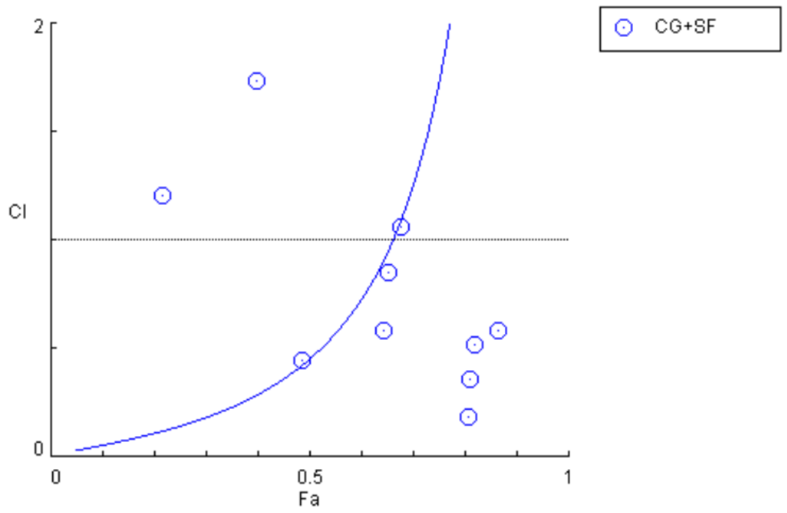

CGEtOAc (μg/mL)	Sorafenib (μM)	CI
200	2	1.20
400	2	2.46
600	2	1.73
800	2	2.18
200	4	0.45
400	4	0.58
600	4	0.85
800	4	1.06
200	8	0.18
400	8	0.36
600	8	0.52
800	8	0.58
