# Supplementary material for: Combination of ethyl acetate fraction from Calotropis gigantea stem bark and sorafenib induces apoptosis in HepG2 cells
Source: PLoS One. 2024 Mar 25;19(3):e0300051. doi: 10.1371/journal.pone.0300051 (PMC10962855; doi:10.1371/journal.pone.0300051)

Supporting information

S4 Fig. The migration rate of HepG2 cells treated with CGEtOAc at 400 μg/mL and sorafenib at 4 μM, both singly and in combination, was evaluated using a wound healing assay after 0-72 h of incubation and compared to the vehicle group. Cells treated with 0.8% DMSO represented the vehicle control. The significant differences in data, presented as the mean ± SD from at least three different experiments, were investigated with a one-way ANOVA using Tukey's HSD test: [a]; $p < 0.05$ compared to 24 h of incubation in the vehicle group, [b]; $p < 0.05$ compared to 48 h of incubation in the vehicle group, and [c]; $p < 0.05$ compared to 72 h of incubation in the vehicle group.

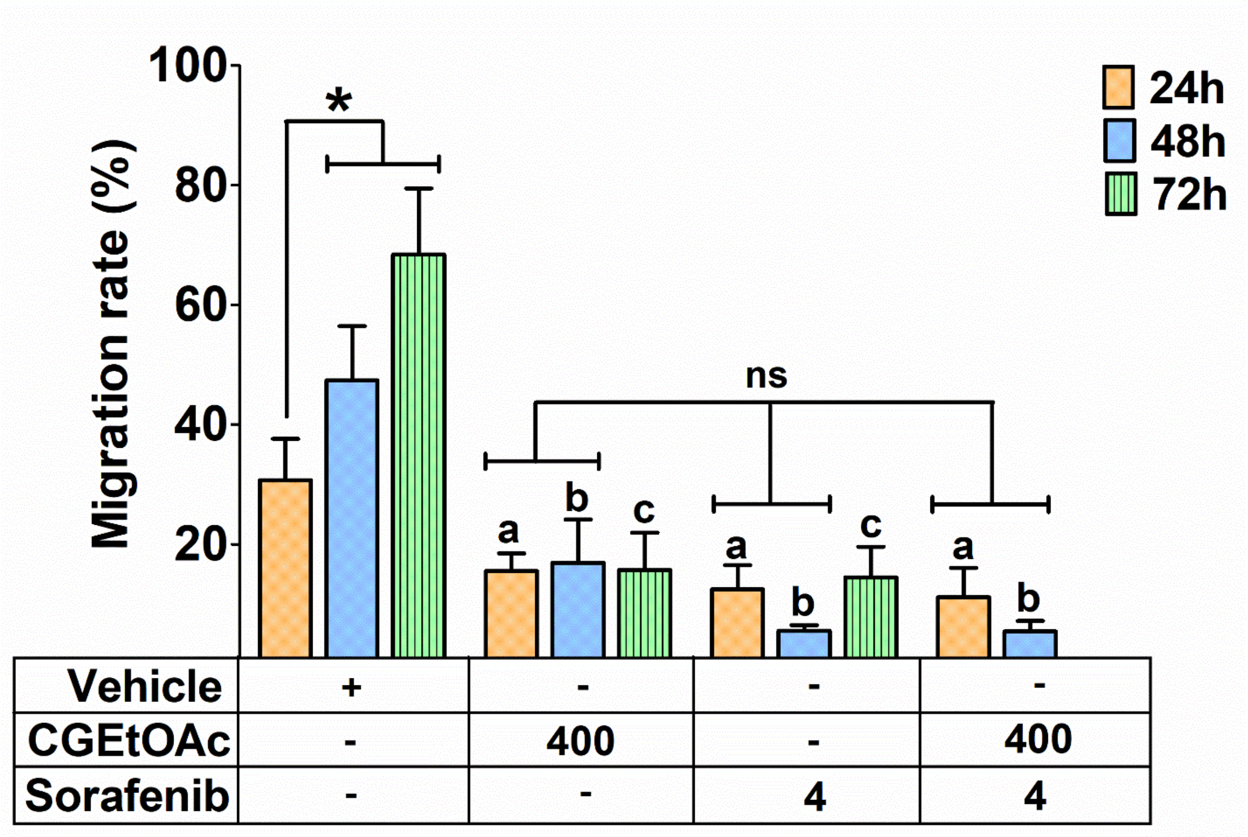

Supplement: S4 Fig — Cells treated with 0.8% DMSO represented the vehicle control. The significant differences in data, presented as the mean ± SD from at least three different experiments, were investigated with a one-way ANOVA using Tukey’s HSD test: a; p < 0.05 compared to 24 h of incubation in the vehicle group, b; p < 0.05 compared to 48 h of incubation in the vehicle group, and c; p < 0.05 compared to 72 h of incubation in the vehicle group. (PDF) [file pone.0300051.s004.pdf]
