# Supplementary material for: Combination of ethyl acetate fraction from Calotropis gigantea stem bark and sorafenib induces apoptosis in HepG2 cells
Source: PLoS One. 2024 Mar 25;19(3):e0300051. doi: 10.1371/journal.pone.0300051 (PMC10962855; doi:10.1371/journal.pone.0300051)

Supporting information

S5 Raw images displaying the gating strategies used in flow cytometry of annexin V and propidium iodide (PI) staining in HepG2 cells after 24 h of incubation, with a combination of 400 μg/mL CGEtOAc and 4 μM sorafenib.

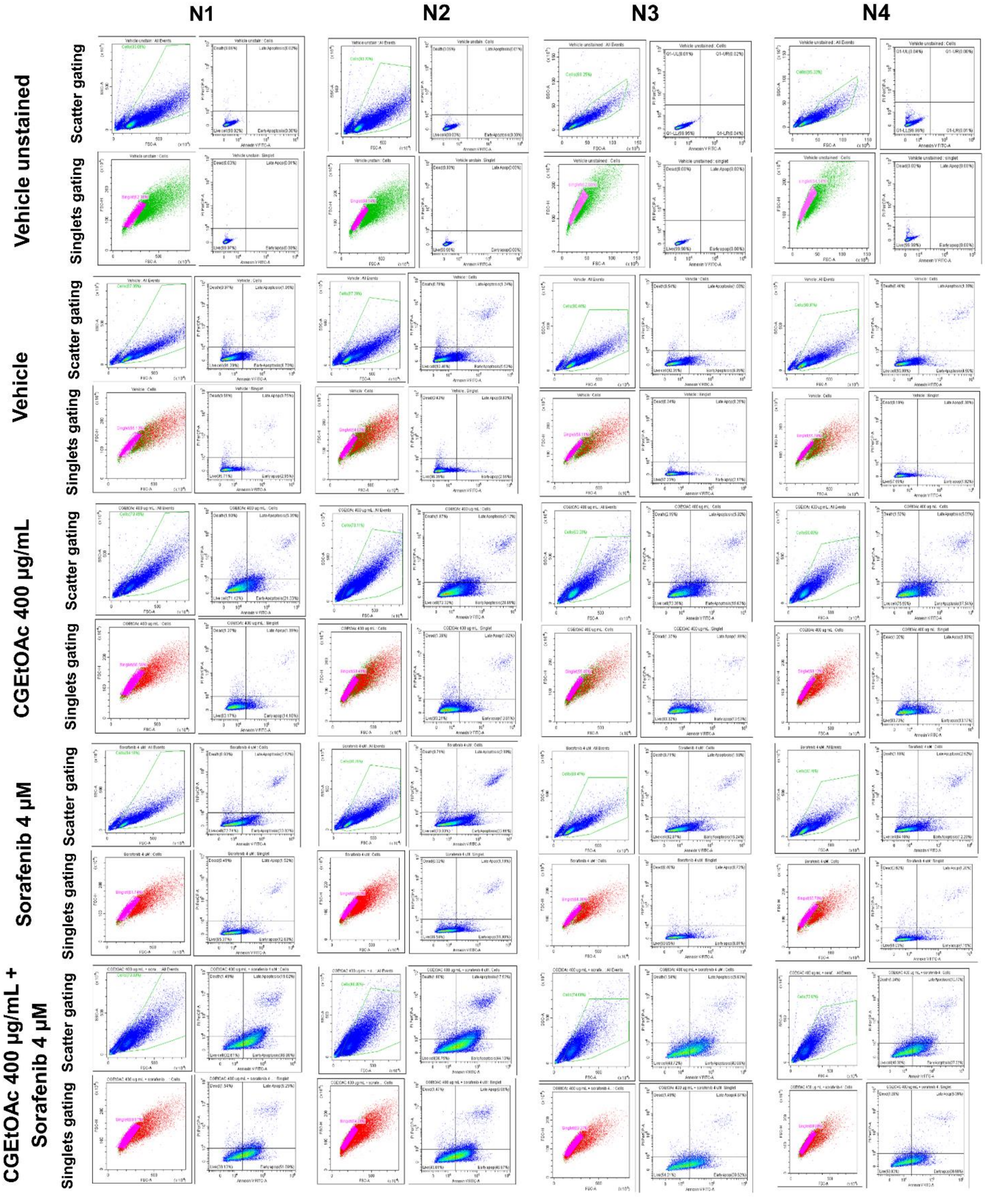

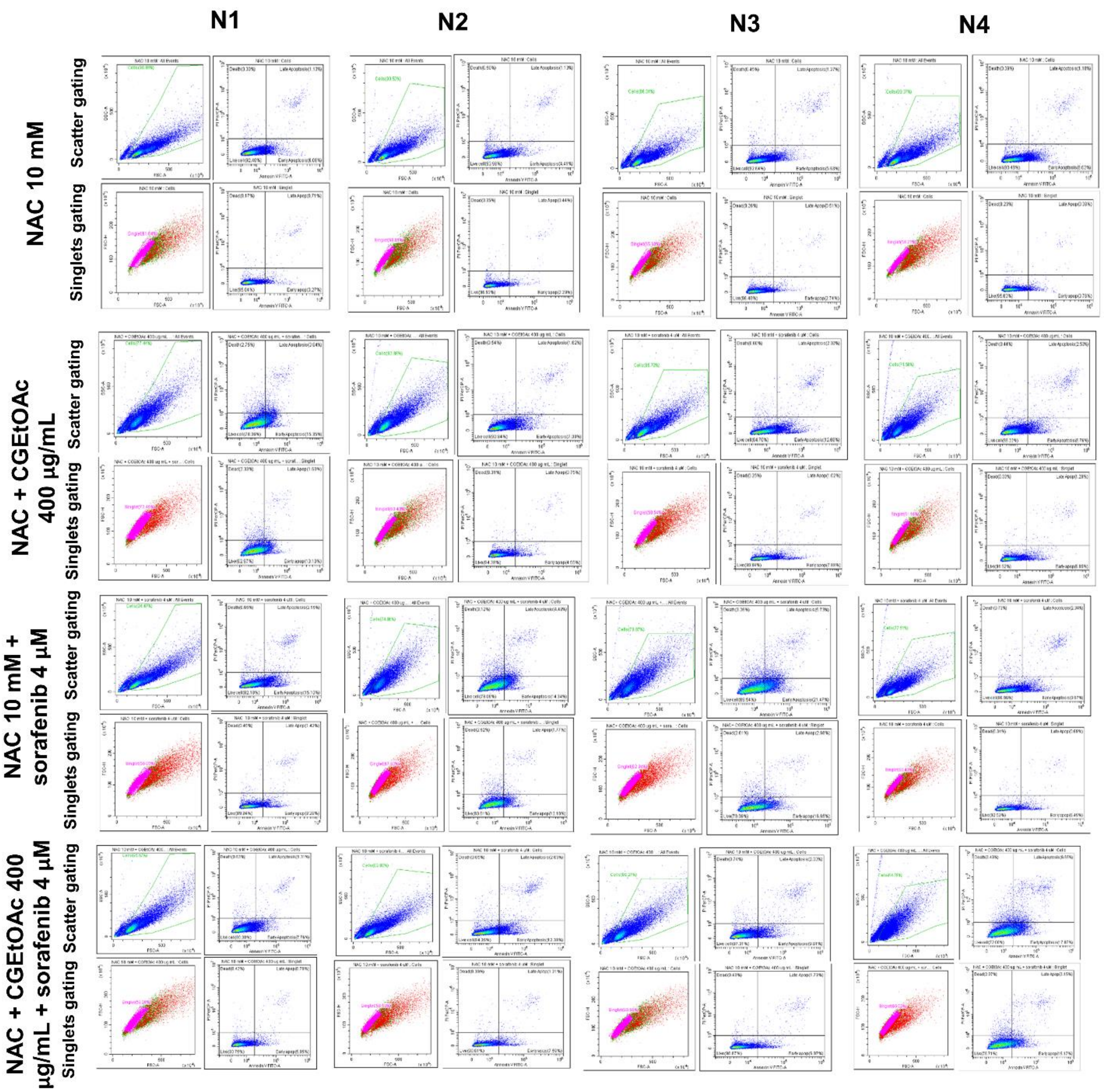

Apoptosis rate by scatter gating

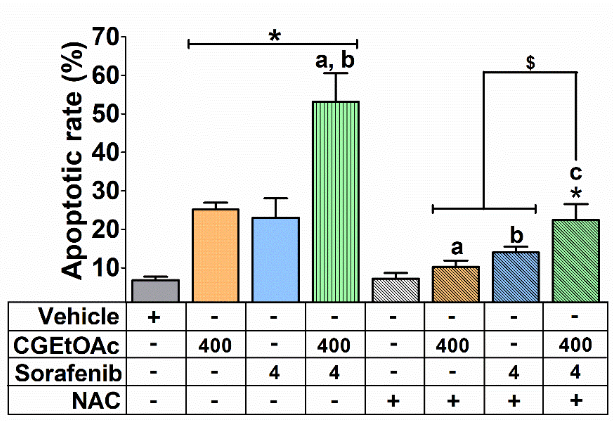

Apoptosis rate by singlets gating

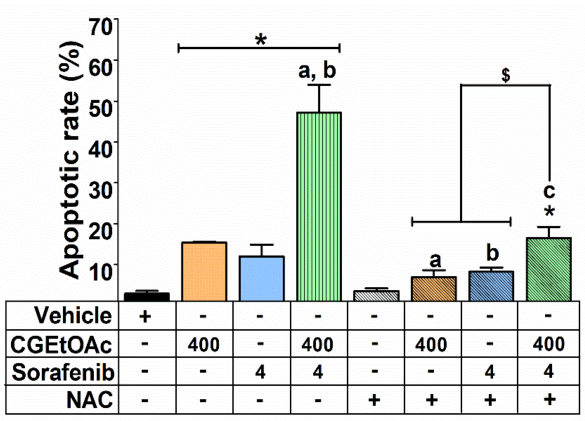

The gating strategy of flow cytometry of annexin V and PI

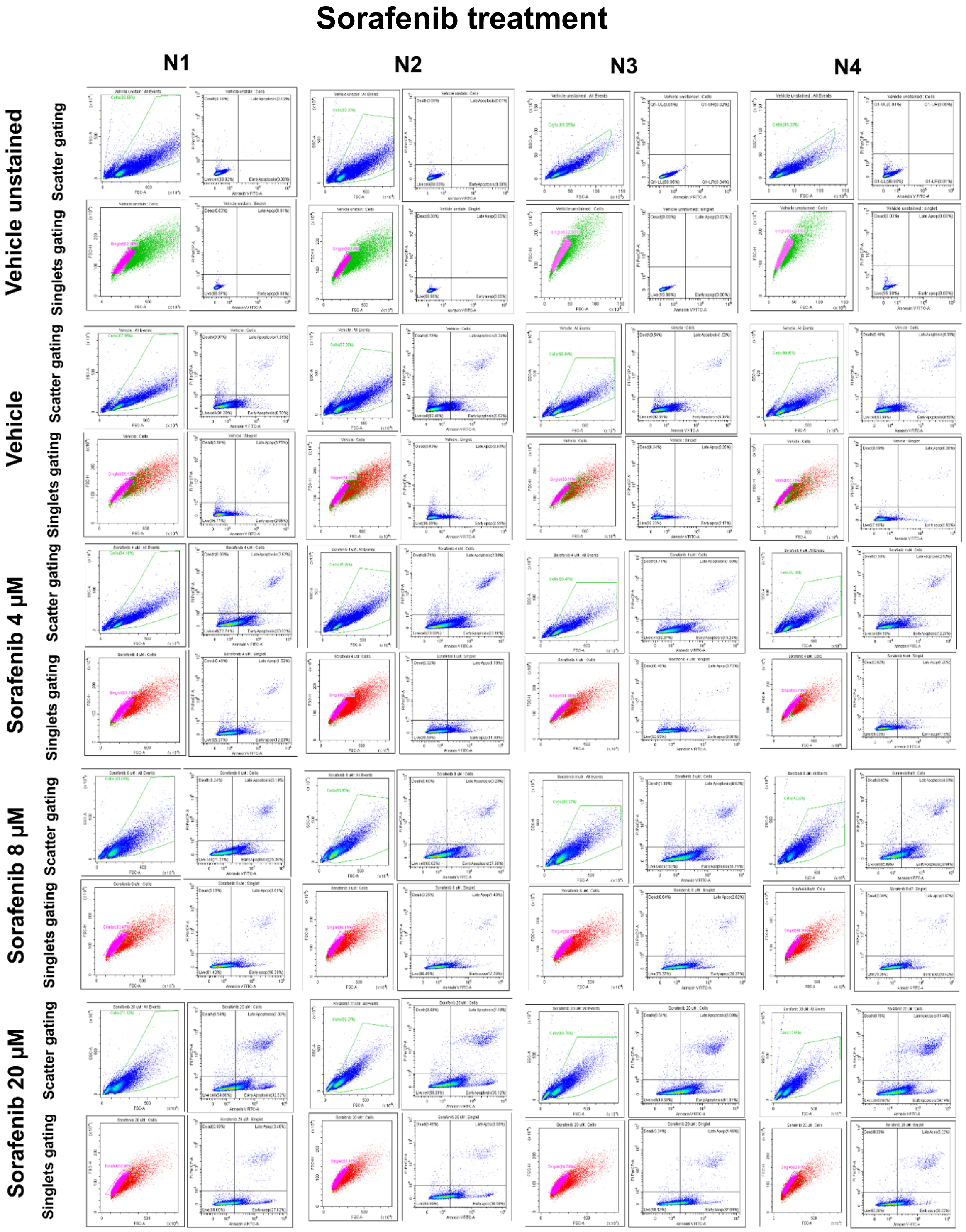

Apoptosis rate of sorafenib treatment by scatter gating

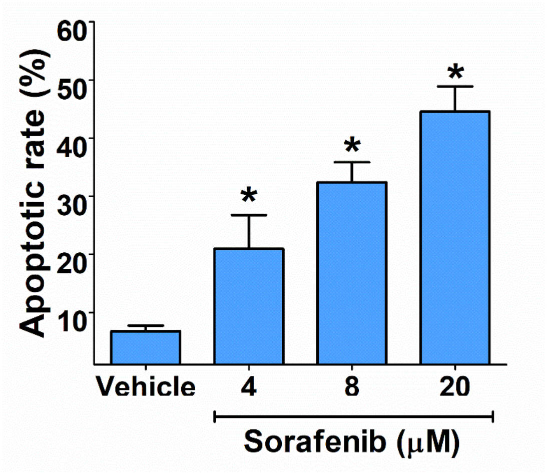

Apoptosis rate of sorafenib treatment by singlets gating

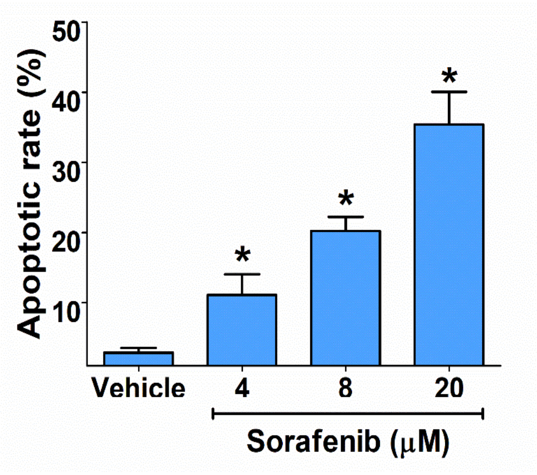

Flow cytometry setting

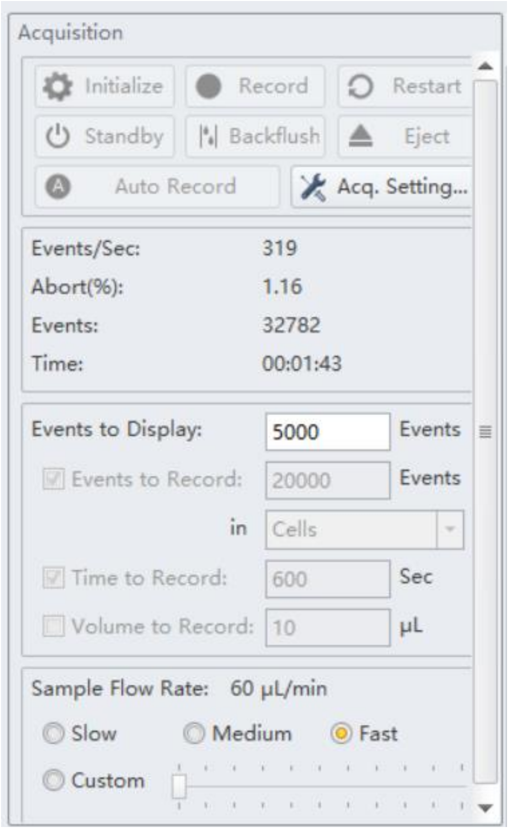

Supplement: S1 Raw images — (PDF) [file pone.0300051.s005.pdf]
