# Supplementary material for: Combination of ethyl acetate fraction from Calotropis gigantea stem bark and sorafenib induces apoptosis in HepG2 cells
Source: PLoS One. 2024 Mar 25;19(3):e0300051. doi: 10.1371/journal.pone.0300051 (PMC10962855; doi:10.1371/journal.pone.0300051)

Supporting information

S6 Raw images of the original uncropped and unadjusted western blot images for HepG2 cells treated with a combination of 400 µg/mL CGEtOAc and 4 µM sorafenib for a 24-h incubation period.

PageRuler™ Prestained Protein Ladder, 10 to 180 kDa #26616

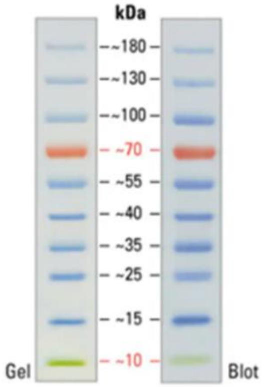

BIO-HELIX - PMB01-0500 / PM001-0500 Blu10 Plus Prestained Protein Ladder / BLUltra Prestained Protein Ladder (6.5 to 270 kDa)

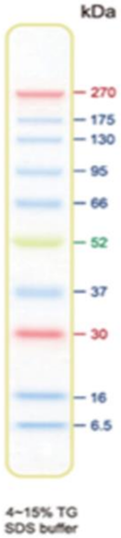

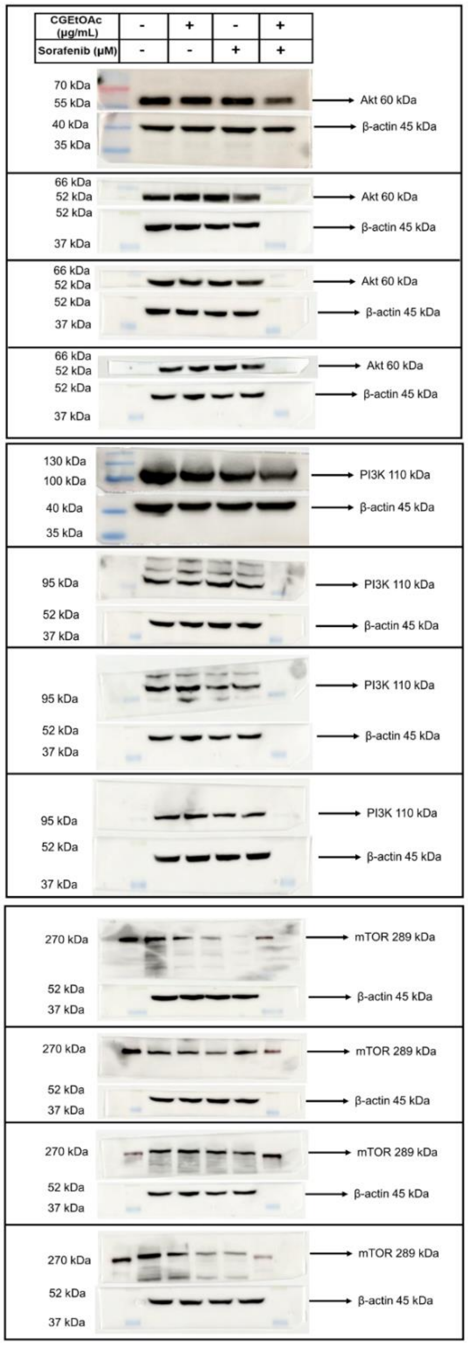

Supplement: S2 Raw images — (PDF) [file pone.0300051.s006.pdf]
