# Supplementary material for: Combination of ethyl acetate fraction from Calotropis gigantea stem bark and sorafenib induces apoptosis in HepG2 cells
Source: PLoS One. 2024 Mar 25;19(3):e0300051. doi: 10.1371/journal.pone.0300051 (PMC10962855; doi:10.1371/journal.pone.0300051)
Supplement: Attachment — Submitted filename: Response to Reviewers.docx [file pone.0300051.s008.docx]

**Reviewers' comments:**

Reviewer's Responses to Questions

**Comments to the Author**

1. If the authors have adequately addressed your comments raised in a previous round of review and you feel that this manuscript is now acceptable for publication, you may indicate that here to bypass the “Comments to the Author” section, enter your conflict of interest statement in the “Confidential to Editor” section, and submit your "Accept" recommendation.

Reviewer #2: (No Response)

Reviewer #3: All comments have been addressed

2. Is the manuscript technically sound, and do the data support the conclusions?

The manuscript must describe a technically sound piece of scientific research with data that supports the conclusions. Experiments must have been conducted rigorously, with appropriate controls, replication, and sample sizes. The conclusions must be drawn appropriately based on the data presented.

Reviewer #2: Partly

Reviewer #3: Yes

3. Has the statistical analysis been performed appropriately and rigorously?

Reviewer #2: Yes

Reviewer #3: Yes

4. Have the authors made all data underlying the findings in their manuscript fully available?

The [PLOS Data policy](http://www.plosone.org/static/policies.action#sharing) requires authors to make all data underlying the findings described in their manuscript fully available without restriction, with rare exception (please refer to the Data Availability Statement in the manuscript PDF file). The data should be provided as part of the manuscript or its supporting information, or deposited to a public repository. For example, in addition to summary statistics, the data points behind means, medians and variance measures should be available. If there are restrictions on publicly sharing data—e.g. participant privacy or use of data from a third party—those must be specified.

Reviewer #2: Yes

Reviewer #3: Yes

5. Is the manuscript presented in an intelligible fashion and written in standard English?

PLOS ONE does not copyedit accepted manuscripts, so the language in submitted articles must be clear, correct, and unambiguous. Any typographical or grammatical errors should be corrected at revision, so please note any specific errors here.

Reviewer #2: Yes

Reviewer #3: Yes

6. Review Comments to the Author

**Reviewer #2:**

In this study conducted by Chaisupasakul and colleagues, the researchers explore the therapeutic potential of the ethyl acetate fraction derived from the stem bark extract of Calotropis gigantea. The focus is on its synergistic effects in combination with sorafenib against HepG2 cells. The research findings demonstrate that the combined application of the extract and sorafenib leads to early apoptosis. The study delves into potential underlying mechanisms, including the examination of factors such as reactive oxygen species (ROS) production and the inhibition of the PI3K/Akt/mTOR pathway. The manuscript has greatly improved since the first submission.

However, problems with the determination of the IC50 is an issue because of the lack of data points towards the bottom of the asymptotic curve used to compute for the IC50. This would in turn, have a cascading effect on the downstream experiments presented since the assessment whether the effect is synergistic or additive would be hard to determine. Further suggestions are written below.

1) In the abstract, “The cytotoxicity of the ethyl acetate fraction of the Calotropis gigantea (L.) Dryand. (C.gigantea) stem bark extract (CGEtOAc) of has been demonstrated in many types of cancers.” should be changed to “The cytotoxicity of the ethyl acetate fraction of the Calotropis gigantea (L.) Dryand. (C.gigantea) stem bark extract (CGEtOAc) has been demonstrated in many types of cancers.”.

**Response:**

We appreciate the positive comments from the reviewer and thank you for these suggestions.

The revised manuscript has been edited as suggested.

2) In the Materials section of the Methodology, there should be a comma after (DMEM)(Corning, USA) instead of a period.

3) Include catalog numbers of FBS, DMEM, MTT, and Lumina Forte Western HRP Substrate.

**Response:**

Thank you for these suggestions.

The revised manuscript has been edited as suggested.

4) “The ultrasonic-assisted extraction with 95% ethanol was used to prepare the crude extract before fractionation using the liquid-liquid chromatography of water and dichloromethane, followed by the rest of the water layer and ethyl acetate.” should be “The ultrasonic-assisted extraction with 95% ethanol was used to prepare the crude extract before fractionation using liquid-liquid chromatography of water and dichloromethane, followed by the rest of the water layer and ethyl acetate.”

**Response:**

Thank you for these suggestions.

The revised manuscript has been edited as suggested.

5) The 50 in IC50 should be a subscript.

**Response:**

Thank you for these suggestions.

The revised manuscript has been edited as suggested.

6) Graph Prism version 9 should be changed to GraphPad Prism version 9.

**Response:**

Thank you for these suggestions.

The revised manuscript has been edited as suggested.

7) “The scratch wounds were consistently in size in each well to avoid variations between conditions” should be “The scratch wounds were consistent in size in each well to avoid variations between conditions”

**Response:**

Thank you for these suggestions.

The revised manuscript has been edited as suggested.

8) There should be no indentation before HepG2 cells in the Fluorescence labeling with JC-1 to evaluate mitochondrial membrane potential section of the Methodology.

**Response:**

Thank you for these suggestions.

The revised manuscript has been edited as suggested.

9) 5-Diphenyltetrazolium Bromide should be 5-diphenyltetrazolium bromide.

**Response:**

Thank you for these suggestions.

The revised manuscript has been edited as suggested.

10) In S2 Fig, adjust the graph such that the origin would meet at y=0 and x=-2.0.

**Response:**

Thank you for these suggestions.

The S2 Fig. has been revised as suggested and is shown below.

S2 Fig.-revised

**S2 Fig. The IC_50_ curves for (A) CGEtOAc and (B) sorafenib in HepG2 cells, and (C) CGEtOAc in IMR-90 cells for 24 h of incubation.**

11) Make sure that all supplementary figures are titled properly just like how you would normally title a main text figure.

**Response:**

Thank you for these suggestions.

The revised manuscript and all figures have been edited as suggested.

12) There is a major concern on how the effectivity of CGEtOAc is and the how the CGEtOAc and sorafenib are set up. The asymptotic curve used to determine the IC50 of the extract and the compound is missing the flat slope to the right (observations with 0% inhibition), hence the accuracy of the IC50 evaluation is compromised since the formula from GraphPad would have no bottom response to be used. This could potentially cause a cascade effect since the next experiments were based off of the computed CIs with the IC50 of CGEtOAc and sorafenib as a baseline.

**Response:**

Thank you for these suggestions. We appreciate the reviewer’s insights.

We have revised cell viability test to enhance the accuracy and reliability of IC_50_ value (Figure 1-revised and S2 Fig-revised with correction of Y-axis name to “% cell viability”). The revised manuscript has been edited as follows.

CGEtOAc was tested at concentrations ranging from 0 to 2,000 µg/mL, while sorafenib concentrations ranged from 0 to 40 µM, as illustrated in Figure 1 and S2 Fig. The cytotoxic effect revealed that cell viability decreased to approximately 18% at CGEtOAc 2,000 µg/mL, with an IC_50_ value of 707.87±49.05 µg/mL. The IC_50_ of CGEtOAc from 0 to 1,000 µg/mL was 771.00 ± 69.74 µg/mL. Additionally, for sorafenib concentrations ranging from 0 to 40 µM, which also exhibited a decrease in cell viability to approximately 10%, the IC_50_ was 8.65±0.33 µM, and at 0 to 20 µM, it was 8.64 ± 0.32 µM, demonstrating a negligible difference.

However, concerning the reviewer’s concern about the missing the flat slope to the right of the asymptotic curve used to determine the IC_50_ of the extract and sorafenib, it’s important to note that the potentially cascade effect on the next experiments was not affected. This is because the cytotoxic effect of CGEtOAc and sorafenib revealed a decrease in cell viability to less than 10-20%. Additionally, in the consequence experiments aimed at evaluating the combination effect, we utilized sub-IC50 concentrations and those around IC50 for both CGEtOAc (at 200,400, 600, and 800 µg/mL) and sorafenib (at 2, 4, and 8 µM). The objective was to employ concentrations as low as possible to generate the combination effect of CGEtOAc and sorafenib.

Additionally, the absence of a flat slope reaching 0% cell viability was due to the limited solubility of CGEtOAc extracts at doses higher than 2,000 µg/mL, which we preliminarily explored. Therefore, the present study restricted the maximum dose of CGEtOAc to 2,000 µg/mL. Concerning the IC_50_ of sorafenib, we consistently obtained IC_50_ values (8.65±0.33 µM for concentrations from 0 to 40 µM) comparable to those reported in other publication (<https://doi.org/10.21873/invivo.13190>, <https://doi.org/10.2147/DDDT.S344750> ). Consequently, the combination effect of CGEtOAc and sorafenib, as demonstrated in Figure 1C, where CGEtOAc was at 400 µg/mL and sorafenib at 4 µM, was deemed suitable for the subsequent experiments and potential future applications in treatment.

Figure 1-revised is shown below.

**Fig 1. Cytotoxicity of** **the ethyl acetate fraction of *C. gigantea* stem bark extracts (CGEtOAc) and sorafenib against HepG2 cells.** The 3-(4, 5-Dimethylthiazol-2-yl)-2, 5-diphenyltetrazolium bromide (MTT) technique was used to assess the cell viability after being treated for 24 h with (A) CGEtOAc, (B) sorafenib, and (C) their combination, demonstrated in the bar graph and (D) heat map analysis. (E) CGEtOAc and (F) the combination of CGEtOAc and sorafenib treatment on IMR-90 cells. Cells treated with 0.8% dimethyl sulfoxide (DMSO) represented the vehicle control. The significant differences in data, presented as the mean ± standard deviation (SD) from at least three different experiments, were investigated with a one-way analysis of variance (ANOVA) using Tukey's honestly significant difference (HSD) test: *; p < 0.05 vs the vehicle control, ^a^; p < 0.05 vs a single CGEtOAc treatment at their own dose, ^b^; p < 0.05 vs a single sorafenib treatment at their own dose.

13) The subtitle “CGEtOAc in combination with sorafenib inhibited the migration ability of HepG2 cells” should be CGEtOA and sorafenib, singly and in combination inhibited the migration of HepG2 cells”.

**Response:**

Thank you for these suggestions.

The revised manuscript has been edited as suggested.

14) Comple this Figure tile in Figure 2. The migratory capacity of HepG2 cells was assessed using a 72-h.

**Response:**

Thank you for these suggestions.

The revised manuscript has been edited, and the figure title in Figure 2 has been completed as followed:

“**Fig 2. The migratory capacity of HepG2 cells was assessed through a 0-72-h incubation with CGEtOAc at 400 µg/mL and sorafenib at 4 µM, both singly and in combination**.”

15) Why is there no data point for 72h treatment for CGEtOAc and sorafenib combination?

**Response:**

We appreciate the reviewer’s suggestions.

In acknowledgment of your valuable question, we have incorporated this issue into the Results section as follows:

“The migration data for the combination of CGEtOAc and sorafenib at 72 h disappeared due to the complete loss of cell viability. Cells detached from the plate, rendering it impossible to measure the gap between cell scratches, attributed to the toxic effects of the combination treatment.”

16) There should be a T-test comparing the combination group with the single treatment to determine whether the combination is actually acting on the migration rate and not just the individual ability of CGEtOAc and sorafenib. An experiment with lower doses combination can be done to determine if it is truly the combination of the two that is inhibiting migration.

**Response:**

We appreciate the reviewer’s suggestions.

The revised Figure 2 and S4 Fig. have been updated to include comparisons between the combination group with the single treatment, as stated as below:

“At a concentration of 400 µg/mL for CGEtOAc, 4 µM for sorafenib, and their combination, cell migration was significantly inhibited. This was demonstrated by a higher percentage of gap distance in a wound healing assay compared to each hour of incubation in the vehicle group, when 0 h set at 100% for each group (Fig 2A and 2B). The percentage of gap distance for the combination of CGEtOAc and sorafenib in each incubation period did not differ from the single treatments. However, the migration data for the combination of CGEtOAc and sorafenib at 72 h disappeared due to the complete loss of cell viability. Cells detached from the plate, rendering it impossible to measure the gap between cell scratches, attributed to the toxic effects of the combination treatment.

The migration rate, as shown in S4 Fig, confirmed a significant inhibition of HepG2 cell migration by CGEtOAc at 400 µg/mL, sorafenib at 4 µM, and their combination compared to each hour of incubation in the vehicle group. The migration rate for the combination of CGEtOAc and sorafenib in each incubation period did not differ from the single treatments.

Our findings indicate that CGEtOAc, sorafenib, and their combination upregulated the anti-proliferative and anti-migratory activities in HepG2 cells.”

Notably, suppression of migration was observed within 24 hours after incubation with CGEtOAc at 400 µg/mL, sorafenib at 4 µM, and their combination, with no significant difference among the 24-, 48- and 72-hour incubation periods. This finding aligns with reports demonstrating that the inhibition of migration remains consistent across different incubation times at a particular concentration of the agent that significant exhibited cytotoxic effects on cancer cells (<https://doi.org/10.21873/anticanres.15241> , <https://doi.org/10.1093/gastro/goaa072> ).

In addition, we found that the suppression of migration by the combination of CGEtOAc and sorafenib did not differ from that observed with their respective single treatments. However, cell viability showed a greater reduction in the combination group compared to each single treatment. Our findings are consistent with report demonstrating that the combination of the chemotherapeutic agent docetaxel and plant extract had a similar inhibitory effect on migration compared to single treatments, as evaluated by measuring gap width in breast cancer cells at every incubation time (<https://doi.org/10.1155/2021/5517944> ). These findings suggest that cell migration was inhibited prior to cell death. After the first 24 hours post-treatment with both lethal and non-lethal doses of radiation and chemotherapy drug paclitaxel, migration of glioblastomas was inhibited prior to cell death. This suggestion proposed that cell migration should be a therapeutic target in anti-metastasis/anti-invasion strategies for improve cancer therapeutic outcomes (<https://doi.org/10.1016%2Fj.bbrep.2021.101071> ).

Thus, our manuscript suggests that each single treatment, CGEtOAc and sorafenib, possesses migration inhibition properties, which may contribute to enhancing the synergistic effect of the combination treatment on anticancer efficiency.

The study evaluated the effects of CGEtOAc at 400 µg/mL, sorafenib at 4 µM, and their combination on the inhibition of cell migration. The results from both MTT and combination index analysis indicated that the combination of CGEtOAc at 400 µg/mL with sorafenib at 4 µM exhibited a combination index (CI) value of 0.5, correlated with an inhibition rate of approximately 60%, suggestive of a synergistic effect. However, combinations with lower concentrations, such as CGEtOAc at 200 µg/mL and sorafenib at 4 mM also yielded a CI of 0.5, albeit with a lesser inhibition rate of 40%. Therefore, we concluded that CGEtOAc at 400 µg/mL and sorafenib at 4 µM were selected for the further experimentation.

Below are the revised versions of Figure 2 and S4 Fig.

Figure 2-revised

**Fig 2.** **The migratory capacity of HepG2 cells was assessed through a 0-72-h incubation with CGEtOAc at 400 µg/mL and sorafenib at 4 µM, both singly and in combination**. (A) Wound healing images were captured with a magnification bar of 500 µm. (B) The percentage of the gap was depicted as a bar graph. Cells treated with 0.8% DMSO represented the vehicle control. The significant differences in data, presented as the mean ± SD from at least three different experiments were investigated with a one-way ANOVA using Tukey's HSD test: *; p < 0.05 vs the 0 h of the vehicle control, a; p < 0.05 compared to 24 h of incubation in the vehicle group, b; p < 0.05 compared to 48 h of incubation in the vehicle group, and c; p < 0.05 compared to 72 h of incubation in the vehicle group, with 0 hours set at 100% for each group.

S4 Fig-revised

**S4 Fig. The migration rate of HepG2 cells treated with CGEtOAc at 400 µg/mL and sorafenib at 4 µM, both singly and in combination, was evaluated using a wound healing assay after 0-72 h of incubation and compared to the vehicle group.** Cells treated with 0.8% DMSO represented the vehicle control. The significant differences in data, presented as the mean ± SD from at least three different experiments were investigated with a one-way ANOVA using Tukey's HSD test: a; p < 0.05 compared to 24 h of incubation in the vehicle group, b; p < 0.05 compared to 48 h of incubation in the vehicle group, and c; p < 0.05 compared to 72 h of incubation in the vehicle group.

17) Fix S4 Fig title as it doesn’t only show 24 h of incubation and not just the combination treatment. This applies to all the Figure titles, as they should be complete and should completely describe the data presented.

**Response:**

We appreciate the reviewer’s suggestions.

This title has been revised and changed to

“**S4 Fig. The migration rate of HepG2 cells treated with CGEtOAc at 400 µg/mL and sorafenib at 4 µM, both singly and in combination, was evaluated using a wound healing assay after 0-72 h of incubation and compared to the vehicle group**.”

18) Define MMP.

**Response:**

We appreciate the reviewer’s suggestions.

The revised manuscript has been edited to address the issue of “mitochondrial membrane potential (MMP)”, as suggested.

19) Figure 3 title should be the apoptotic effect of CGEtOAc, sorafenib, and their combination against HepG2 cells after exposure for 24h.

**Response:**

Thank you for these suggestions.

The revised manuscript has been edited as suggested.

20) “As shown in Fig 5A, 400 CGEtOAc μg/mL combined with 4 μM sorafenib upregulated ROS production with the green fluorescence intensity of dichlorofluorescein compared to the vehicle control and their respective treatment groups.” should be “As shown in Fig 5A, 400 μg/mL CGEtOAc combined with 4 μM sorafenib upregulated ROS production with the green fluorescence intensity of dichlorofluorescein compared to the vehicle control and their respective treatment groups.”

**Response:**

Thank you for these suggestions.

The revised manuscript has been edited as suggested.

21) The subtitle “The apoptotic response following treatment with a combination of CGEtOAc and sorafenib involved the inhibition of the PI3K/Akt/mTOR pathway in HepG2 cells” should be “The apoptotic response following treatment with a combination of CGEtOAc and sorafenib may involve the inhibition of the PI3K/Akt/mTOR pathway in HepG2 cells” since the blot experiments did not show whether inhibition of PI3K/Akt/mTor by CGEtoAc and sorafenib and their combination is happening dependently or independently of apoptosis.

**Response:**

Thank you for these suggestions.

The revised manuscript has been edited as suggested.

22) “The ethyl acetate fraction contains the highest levels of cardiac glycosides and phenolics, however, triterpenoids, flavonoids, and calotropins are present in lover levels than dichloromethane and other extract fractions.” should be “The ethyl acetate fraction contains the highest levels of cardiac glycosides and phenolics, however, triterpenoids, flavonoids, and calotropins are present in lower levels than dichloromethane and other extract fractions.”

**Response:**

Thank you for these suggestions.

The revised manuscript has been edited as suggested.

23) Make sure that all compounds that are written in the article are in lower case (i.e. lanatoside C, raddeanin A, kaemperol, etc.) since these chemical compounds are not proper nouns.

**Response:**

We appreciate the reviewer's suggestions.

The revised manuscript has been carefully reviewed and edited as suggested.

24) The statement “Inhibition of PI3K/Akt/mTOR expression was also demonstrated as a mediator of apoptosis following combination therapy.” should be rewritten as a hypothesis or a possibility as the experiments presented are not a direct evidence of its association with apoptosis.

**Response:**

Thank you for these suggestions.

The revised manuscript has been edited as follows, incorporating your recommendations.

“Inhibition of PI3K/Akt/mTOR expression also supports the hypothesis that the apoptotic mechanism of CGEtOAc, when combined with sorafenib, may be associated with the downregulation of the upstream PI3K/AKT/mTOR signaling cascade pathway. However, the experiments presented did not provide sufficient evidence of this inhibition in a direct association with the apoptotic effects following the treatment.”

We would like to inform you that our manuscript's title has been edited using “ethyl acetate” to replace “Ethyl acetate”, thus the title is “Combination of ethyl acetate fraction from *Calotropis gigantea* stem bark and sorafenib induces apoptosis in HepG2 cells".

In conclusion, we sincerely thank the editor of the PLOS ONE and the reviewers for their insightful comments and suggestions. We appreciate the time and effort you have dedicated to assisting our work to be suitable for publication. We have carefully noted all your suggestions and comments and have addressed them, as they are beneficial for strengthening this study.

Sincerely yours,

Corresponding authors

**Reviewer #3:**

The revised version of this manuscript from Chaisupasakul et al. has improved considerably from its previous version. However, there are some minor suggestions in the discussion section that need to be addressed before recommending it for publication.

1. The authors have mentioned the possible role of cardiac glycoside in conferring anticancer properties, and so based on their findings in HPLC-ESI-MS, the line in discussion “the cardiac glycoside composition in the ethyl acetate fraction of the C. gigantea stem bark extract may be postulated to be one of the key components that confer anticancer properties to the ethyl acetate fraction when combined with sorafenib in HepG2 cells” should be modified accordingly.

**Response:**

We appreciate the reviewer's suggestions.

The revised manuscript has been carefully reviewed and edited incorporating the suggested changes as follows:

“Consequently, based on the findings in HPLC-EIS-MS, it may be postulated that the cardiac glycoside composition in the ethyl acetate fraction of the *C. gigantea* stem bark extract is one of the key components that confer anticancer properties to the ethyl acetate fraction when combined with sorafenib in HepG2 cells.”

2. The present study reports exposure to CGEtOAc and sorafenib reduces the expression of PI3K/Akt/mTOR. In contrast, previous studies from Dai et al. 2018 and Wang et al. 2020 have shown reduced activation of PI3K/Akt/mTOR signaling pathways when sorafenib was given with other combinations. Does CGEtOAc has any role in the transcription of PI3K/Akt/mTOR? Are there any available studies showing transcription regulation of PI3K/Akt/mTOR from any other plant extract?

**Response:**

We acknowledge the reviewer’s comments regarding the reduced activation of PI3K/Akt/mTOR signaling pathways instead of the transcription of protein expression following various treatments with plant extracts, sorafenib, both individually and in combination. However, it is worth noting that several reports have demonstrated the effects of plant extracts, sorafenib, and their combination on the transcription of protein signaling pathway expression in cancer cells. However, there have been no reports on the effect of *C. gigantea* extracts, either alone or in combination with sorafenib, on the inhibition of PI3K/Akt/mTOR expression in cancer cells. The following publications serve as examples of *C. procera* (the family Apocynaceae) extracts and cardiac glycosides in the inhibition of the expression of the PI3K/AKT/mTOR pathway in cancer cells.

The leaves of *C. procera* extracts exhibited inhibition on AKT/mTOR expression, inducing apoptosis in cancer cells (<https://doi.org/10.1016/j.heliyon.2023.e16706> ). Cardiac glycosides, peruvoside, inhibited the expression of PI3K and mTOR in cancer cells (<https://doi.org/10.1016/j.lfs.2019.117147> ). Cardiac glycoside ouabain exhibited inhibition of the expression of mTOR and p-AKT in cancer cells (<https://doi.org/10.3892/mmr.2018.8587> ). Lanatoside C, the cardiac glycoside, downregulated gene expression of AKT, PI3K, and mTOR in cancer cells (<https://doi.org/10.3390/biom9120792> ). Digoxin decreased PI3K and p-Akt protein expression levels in cancer cells (<https://doi.org/10.1016/j.redox.2019.101131> ). Ouabain significantly decreased the levels of PI3K and p-AKT(Thr308) in cancer cells (<https://doi.org/10.21873/anticanres.15241> ).

Thus, we have addressed the following points in the discussion:

“Inhibition of PI3K/Akt/mTOR expression also supports the hypothesis that the apoptotic mechanism of CGEtOAc, when combined with sorafenib, may be associated with the downregulation of the upstream PI3K/AKT/mTOR signaling cascade pathway. However, the experiments presented did not provide sufficient evidence of this inhibition in direct association with the apoptotic effects following the treatment. Furthermore, consistent with our findings, there have been reports on the effect of *C. procera* extracts and cardiac glycosides in the inhibition of the expression of the PI3K/AKT/mTOR pathway in correlation to induce apoptosis cancer cells (Ref: ).”

The following publications demonstrate examples of reports revealing the effects of plant extracts on the inhibition of the expression of the PI3K/AKT/mTOR pathway in cancer cells.

Matrine (MAT), a compound extracted from *Sophora flavescens* Aiton, exhibited anticancer against breast cancer cells in a 24-h incubation by inhibiting the expression of PI3K/AKT pathway (<https://doi.org/10.1038%2Fs41598-023-39655-9> ).

The anticancer activity against renal cell carcinoma of gypenosides of *Gynostemma pentaphylla* (Thunb.) Makino was demonstrated through the downregulation of mRNA and protein expressions of the AKT/P-AKT-mTOR-P-mTOR pathway (<https://doi.org/10.1016/j.jep.2021.113907> ).

Bupleurum, the dried root of *Bupleurum chinensis* DC. downregulates both the genes and protein expressions of PI3K, Akt, mTOR and phosphorylated proteins P-PI3K, P-Akt, P-MTOR (<https://doi.org/10.1016/j.jep.2021.114742> ), along with other publications including *Celastrus orbiculatus* extracts ((<http://dx.doi.org/10.2174/1871520619666190731162722>), *Scutellaria barbata* D.Don (<https://doi.org/10.3892/or.2017.5892> ).

Additionally, anticancer drugs have been reported to exhibit potential anticancer effects through the inhibition of protein expressions in PI3K/Akt/mTOR signaling pathways. Sorafenib has been shown to suppress PI3K expression (<https://doi.org/10.3389/fonc.2022.852095> , <https://doi.org/10.3892%2Fol.2018.8536>, <http://www.ncbi.nlm.nih.gov/pmc/articles/pmc6789287/>, ), decrease the expression of the PI3K/Akt pathway (doi: 10.1186/s12964-023-01355-2, <https://doi.org/10.18632/oncotarget.9168> ), suppress AKT expression downstream effectors including 4E-BP1 and eIF4E (*DOI:* 10.1097/CAD.0000000000001056), and downregulate the PI3K/AKT/mTOR pathway (PMID: 25778319).

We also addressed in the discussion that there have been reports indicating that sorafenib, in combination with other plant extracts, exhibited suppression of PI3K/Akt/mTOR expression in cancer cells.

“Furthermore, osthole (coumarins), sorafenib, and the combination of both compounds suppressed PI3K and Raf kinases, resulting in cytotoxic effects in cancer cells (<https://doi.org/10.3390/molecules25215192> ). Additionally, sorafenib in combination with capsaicin downregulated the expression levels of Akt, mTOR and p70S6K in LM3 human hepatocellular carcinoma cells, thereby inducing apoptosis (<https://doi.org/10.3892/or.2018.6754>).”

3. In the discussion, the author also should shed lights on other possible mechanism participating in the apoptotic action of the current combination therapy

**Response:**

Thank you for these suggestions, which contribute to strengthening the manuscript's correlation between results and the potential mechanisms underlying the anticancer effect of the combination of CGEtOAc and sorafenib.

This manuscript has provided a discussion into the possible mechanism of HepG2 cells exposed to a combination of CGEtOAc and sorafenib, leading to activation of apoptosis. ROS generation was found to be predominantly responsible for the apoptosis-inducing effects of the combined therapy; however, ROS is not the sole mechanism participating in the apoptotic action. Inhibition of PI3K/Akt/mTOR expression also supports the hypothesis that the apoptotic mechanism of CGEtOAc, when combined with sorafenib, may be associated with the downregulation of the upstream PI3K/AKT/mTOR signaling cascade pathway. Other mechanisms may work in conjunction to generate this treatment efficiency.

We also discussed that ROS production has been reported as a regulator of PI3K/Akt/mTOR expression, which results in cancer cell apoptosis. Furthermore, we discussed sorafenib's mechanism in triggering apoptosis in cancer cells. Moreover, this manuscript addressed the possible mechanism of a combination of sorafenib with anticancer agents, which promotes cancer cell apoptosis.

Regarding the review’s comment that provides strength to the potential discussion of this study, the revised manuscript has addressed the possible mechanism of the anticancer effect of a combination of *C. gigantea* extracts and sorafenib as follows:

“Notably, several studies have reported the apoptotic effect of cardiac glycosides, with this compound identified as one of the major components in *C. gigantea* extracts that exhibit anticancer activity. This effect is achieved through the mechanism of inhibiting Na^+^/K^+^ ATPase, leading to an increase in intracellular Ca^2+^ via the Na^+^/Ca^2+^ exchanger <https://doi.org/10.1007/s10549-005-9053-3> , <https://doi.org/10.1371/journal.pone.0287769> . Cardenolides from *C. gigantea* exhibited potent anticancer effects against breast cancers by inhibiting Na^+^/K^+^ ATPase, resulting in an increase in intracellular Ca^2+^ via the Na^+^/Ca^2+^ exchanger-dependent manner, ultimately leading to apoptosis <https://doi.org/10.1021/acs.jnatprod.0c00423> . Cardenolide UNBS1450, having lower affinity for the α-2 Na^+^/K^+^ ATPase than other cardenolides, induced downregulation of myeloid cell leukemia-1, a member of the anti-apoptotic protein family downstream of Na^+^/K^+^ ATPase activity, contributing to apoptosis in cancer cells <https://doi.org/10.1038/cddis.2015.134> . Various signaling pathways downstream of Na^+^/K^+^ ATPase inhibition has been demonstrated to contribute to the inhibition of cancer cell proliferation. Oleandrin, a cardiac glycoside extracted from the leaves of *Neriaum oleander*, induced accumulation of Ca^2+^, leading to ER stress-mediated cytotoxic effects, enhancing immunogenic cell death in both in vitro and in vivo cancer models <https://doi.org/10.1038/s41419-021-03605-y> . Ascleposide, a natural cardenolide isolated from *Reevesia formosana*, downregulated the oncoprotein c‐Myc protein and inhibited the phosphorylation of tumor suppressor protein Rb, resulting in the inhibition of cancer cell cycle progression and blocking cell proliferation. This effect was attributed to an increase of α‐tubulin acetylation, which contributed to the inhibition of Na^+^/K^+^ ATPase activity by enhancing the endocytosis of this protein in cancer cells through activation of the mitogen‐activated protein kinases (MAPKs) pathway <https://doi.org/10.1002/pros.23944> .

Additionally, toxicarioside G, a cardenolide isolated from *C. gigantea*, exhibited another mechanism of anticancer activity by inhibiting cancer cell viability and proliferation. It enhanced Yes1 associated transcriptional regulator (YAP) dephosphorylation and nuclear localization, along with downstream target gene expression <https://doi.org/10.3892/or.2021.8175> . Calotropin, a cardiac glycoside derived from *C. gigantea*, demonstrates an inhibitory effect on the regulation of metabolic reprogramming in cancer, specifically targeting aerobic glycolysis (the Warburg effect). This leads to activation of cell cycle arrest and inhibition metastasis in cancer cells, ultimately contributing to activation of apoptosis <https://doi.org/10.1016/j.jobcr.2023.09.002>. Another cardiac glycoside, 3′-epi-12β-hydroxyfroside, isolated from the roots of *C. gigantea*, induced autophagy, leading to enhanced apoptosis. This effect was mediated by downregulation of the heat shock protein 90 (Hsp90)-regulated Akt/mTOR pathway in lung cancer cells <https://doi.org/10.7150/thno.23304>. Lanatoside C, a cardiac glycoside, has been shown to induce apoptosis in cancer cells by suppressing the Janus kinase (JAK)/signal transducer and activator of transcription (STAT)/cytokine signaling (SOCS) (JAK2/STAT6/SOCS2) pathway <http://www.ncbi.nlm.nih.gov/pmc/articles/pmc8387865/> , DOI: 10.3892/ol.2021.13001.”

“In addition to the possible mechanism proposed in this study for CGEtOAc, combined with sorafenib-induced apoptosis in cancer cells, which involves increased ROS accumulation and inhibition of PI3K/Akt/mTOR expression, the apoptotic effects resulting from the combination treatment of compounds found in *C. gigantea* extracts and anticancer agents may be attributed to several mechanisms. For instance, the synergistic effect of sorafenib in combination with the Na^+^/K^+^ ATPase inhibitor berbamine, an alkaloid isolated from *Berberis amurensis*, and cardiac glycosides like ouabain, is proposed to be contributed by the potentiation of epidermal growth factor receptor (EGFR)-mediated ERK1/2 and p38MAPK activation by berbamine <https://doi.org/10.1111/bph.15616> . Similarly, the combination of digitoxin and sorafenib has been reported to suppress p-ERK, hypoxia inducible factor (HIF)-1α, HIF-2α, and VEGF expression, contributing to apoptosis in cancer cells <https://doi.org/10.3892/mmr.2016.6096> . Furthermore, the combination of cardenolide derivative, AMANTADIG (3β-[2-(1-amantadine)-1-on-ethylamine]-digitoxigenin) and docetaxel induced apoptosis through the inhibition of surviving protein expression in human androgen-insensitive prostate cancer cells. (<https://doi.org/10.1016/j.biopha.2018.08.028> ).”

We would like to inform you that our manuscript's title has been edited using “ethyl acetate” to replace “Ethyl acetate”, thus the title is “Combination of ethyl acetate fraction from Calotropis gigantea stem bark and sorafenib induces apoptosis in HepG2 cells".

In conclusion, we sincerely thank the editor of PLOS ONE and the reviewers for their insightful comments and suggestions. We appreciate the time and effort you have dedicated to assisting our work to be suitable for publication. We have carefully noted all your suggestions and comments and have addressed them, as they are beneficial for strengthening this study.

Sincerely yours,

Corresponding authors
